# Technological Characterisation of Probiotic Lactic Acid Bacteria as Starter Cultures for Dry Fermented Sausages

**DOI:** 10.3390/foods9050596

**Published:** 2020-05-07

**Authors:** Nadia de L. Agüero, Laureano S. Frizzo, Arthur C. Ouwehand, Gonzalo Aleu, Marcelo R. Rosmini

**Affiliations:** 1 Faculty of Agricultural Sciences, Catholic University of Córdoba, 5000 Cordoba, Argentina; nadiaaguero@hotmail.com (N.d.L.A.); gonzaloaleu@gmail.com (G.A.); 2 Laboratory of Food Analysis “Rodolfo Oscar DALLA SANTINA”, Institute of Veterinary Science (ICiVet Litoral), National University of the Litoral—National Council of Scientific and Technical Research (UNL/CONICET), 3080 Esperanza, Argentina; lfrizzo@fcv.unl.edu.ar; 3 Department of Public Health, Faculty of Veterinary Science, National University of Litoral, 3080 Esperanza, Argentina; 4 Global Health and Nutrition Sciences, DuPont Nutrition and Biosciences, 02460 Kantvik, Finland; arthur.ouwehand@dupont.com

**Keywords:** probiotic, dry fermented sausages, healthy meats, lactobacillus

## Abstract

The objective of this study was to investigate probiotic microorganisms for use as starter cultures in dry fermented sausages production. A total of eight strains were studied evaluating technological and safety characteristics including the ability to grow, lactic acid production, gas formation, catalase activity, nitrate reductase activity, proteolytic activity, lipolytic activity, hydrogen peroxide production, salt tolerance, performance at low temperatures, decarboxylation of amino acids and antimicrobial activity against pathogens associated with the product. *Lactobacillus rhamnosus* R0011, *L. rhamnosus* Lr-32, *Lactobacillus paracasei* Lpc-37, *Lactobacillus casei* Shirota and *Enterococcus faecium* MXVK29 were good candidates for use as fermented sausages starters cultures because they showed the best technological and safety properties since they did not demonstrate amino acid decarboxylation but showed antimicrobial activity against *Listeria monocytogenes*, *Escherichia coli*, *Salmonella Dublin* and *Staphylococcus aureus*. *L. rhamnosus* Lr-32 was the strain best tolerating the levels of salt, nitrate and low pH during the simulated stages of fermentation and ripening of sausage. The strain was thus the most promising of the tested probiotics as sausage starter culture. The findings warrant studies in a meat matrix, such as that of raw-cured sausage, to evaluate the effects of *L. rhamnosus* Lr-32 under actual conditions.

## 1. Introduction

The demand for safe, innovative and healthy food products has been a stimulus for the development of new fermented meat products. Today’s consumer requires that food provides the necessary nutrients and supports health; consequently, meat products with added functional ingredients, or that contain smaller amounts of ingredients that are considered not beneficial for health (salt, fat, etc.) are in greater demand [1,2]. Functional foods are defined as foods that contain some health promoting component beyond traditional nutrients. An example of a functional food is those in which probiotic microorganisms have been incorporated [3].

Probiotics are defined as ‘live microbial food supplements that, when administered in adequate amounts confer a health benefit on the host’ [4]. Such probiotic microorganisms are capable of contributing to the balance of the host’s intestinal microbiota, modulate immune response and can act in various other ways as health promoters [5]. Until now, the most common use of these microorganisms is in fermented dairy products but some authors propose the use of probiotic lactic acid bacteria (LAB) as starter cultures for fermented meat products [2,6,7,8,9,10,11]. The commercialisation of probiotic meat products and commercial application of probiotic microorganisms in fermented sausages is not common yet [12], and in vitro and industrial scale studies are still scarce. The use of probiotic cultures can positively affect the process of fermented sausages, resulting in new technological properties and a beneficial effect on human health. Much of the research is based on the study of bacteria that are commonly associated with the meat environment and which possess the appropriate physiological requirements and health-promoting properties. Such bacteria can be obtained by screening natural sausage or existing commercial meat starter cultures for probiotic properties. Alternatively, the performance of strains with documented health-promoting properties may be investigated in a fermented meat environment [11,13]. Other researchers have studied the use of a mixed starter combining a food starter with a probiotic microorganism [12,14].

LAB as well as Coagulase-Negative Cocci (CNC) are important microorganisms used as starter cultures in fermented meat products [15]. The fermentation improves the quality, safety and stability of the product, extends its shelf life and provides microbial diversity that result in new sensory properties [7,16,17]. The use of probiotic microorganisms as starter cultures could contribute beneficially to the health of the consumer [17]. The development of probiotic starter cultures requires prior knowledge of the microbiota that participates in the spontaneous process and technologically characterise the strains that are intended to be used; in order to select those that present the best technological properties. The fermented sausage is a complex ecological system in which the natural microbiota, together with some raw materials can present adverse conditions for microorganisms incorporated for technological purposes. Thus, probiotic LAB should tolerate the presence of salts, low pH levels and low temperatures [11,15]. 

These microorganisms must be well adapted to the ecological environment and processing conditions of sausages and therefore, be able to develop more efficiently and grow rapidly to compete with the natural microbiota present [18,19]. As a result, they dominate the fermentation process in order to carry out the desired metabolic activities and reach levels that enable the display of health-promoting effects [11]. The enzymatic properties of lipases, proteases, catalases, nitrate and nitrite reductases are very important during the manufacturing process. Some strains of LAB can carry out these functions in the meat matrix which makes them of interest in the development of competitive starter cultures adapted to meat matrices [17,20,21,22]. The tolerance of CNCs, which will be part of the mixed starter culture, should also be studied [17,21,22]. 

Biogenic amines (BA), organic bases with aliphatic, aromatic or heterocyclic structures, are found in various foods and are mainly produced by microbial decarboxylation of amino acids [23,24]. The accumulation of BA in food requires the presence of precursors (amino acids), microorganisms with amino acid decarboxylase activity and favourable conditions for growth and metabolic activity. Such requirements are met during sausage fermentation [21]. A wide variety of meat and meat product LAB can decarboxylate amino acids [21] and BA concentrations can be high enough to cause food poisoning [24,25]. As a result of this, it is necessary to select LAB that do not show amino decarboxylase activity when they are to be used as starter cultures in sausage preparation [26,27].

LAB found in meat may produce a variety of bacteriocins that are generally active against other LAB (which contribute to the competitiveness of the producing strain) and Gram positive pathogens that are transmitted through food, such as *Listeria monocytogenes*, *Staphylococcus aureus*, *Clostridium perfringens* and *Bacillus cereus* [28]. The production of bacteriocins with a wide inhibition range, especially towards foodborne pathogens, combined with acid production and reduction of the batter pH is therefore highly desirable, as this would ensure the competitiveness of the initiating strain while reducing the development of an undesirable microbiota [21]. Probiotic bacteria could be incorporated into the complex meat system and be potentially successful starter cultures. In the process of selecting these probiotic bacteria, properties such as: growth capacity and production of lactic acid at the desired temperatures, tolerance to low pH and high salt concentrations (nitrite and NaCl) and enzymatic and antagonistic activities should be taken into account.

The first stage in the design of a starter culture for a meat product is to characterise the probiotic LAB strains and select the most appropriate [21]. In meat fermentations, the main function of LAB is to obtain a rapid pH drop of the batter. Furthermore, the strains must be able to grow and survive in the conditions that exist in the sausages. 

The objective of this work was to study commercial LAB probiotics and select the most suitable to be used as a starter of a dry fermented sausage, according to its technological characteristics, strain safety and antimicrobial activity against pathogenic microorganisms associated with these foods.

## 2. Materials and Methods 

### 2.1. Sampling

The microorganisms used in the tests are listed in Table 1. Probiotic strains were obtained from Lallemand (Montreal, Canada), DuPont (Madison, WI, USA), Yakult (Tokyo, Japan), BioGaia (Stockholm, Sweden) and the Autonomous Metropolitan University (Distrito Federal, México). *Pediococcus pentosaceus* PCFF-1 and *Staphylococcus xylosus* DD-34 were purchased from Chr. Hansen (Hørsholm, Denmark). Some trials included strains of *Lactobacillus plantarum* and *Lactobacillus sakei* isolated from the indigenous microbiota of sausage (Córdoba, Argentina). *Staphylococcus aureus* and *Listeria monocytogenes* were kindly provided by the Pediatric Hospital of the Infant Jesus (Córdoba, Argentina). *Escherichia coli* and *Salmonella* Dublin belong to the strain collection of the National University of the Litoral (Department of Public Health, National University of the Litoral, Esperanza, Santa Fe, Argentina). The beneficial effects of probiotic strains have been documented among others in the following studies: *L. rhamnosus* R0011 and *L. helveticus* R0052 by Evans et al. [29]; *L. rhamnosus* Lr- 32 by Miyazima et al. [30]; *L. paracasei* Lpc-37 by Ouwehand et al. [31]; *Lactobacillus casei* Shirota by Tripolt et al. [32]; *Enterococcus faecium* MXVK29 by Alvarez-Cisneros et al. [33]; *Lactobacillus reuteri* DSM17938 by Savino et al. [34] and *L. reuteri* DSM17918 by Lin et al. [35].

### 2.2. Quantification of Strains

The strains were inoculated in de Man, Rogosa, Sharpe (MRS) broth (Britania^®^, Buenos Aires, Argentina) and incubated in a thermostatic bath with continuous shaking at 37 °C. Optical density was measured with a wide band filter (OD 600 nm) with an automatic turbidimeter (Bioscreen C, Turku, Finland) and parallel plate counts were performed to determine colony forming units (CFU). This was done at the start (*t* = 0), at 2 h (when turbidity was observed in the tube) and over 12 h (measurements approximately every 30–40 min) until 20 measurements were taken in total. The tests were carried out in triplicate and on two different occasions. For each strain, its standard calibration curve was constructed from the OD 600 obtained and the logarithm of the CFU at 37 °C; we assumed no influence of cultivation temperature on the calibration curve. The curves were used to assess the ability of the strains to grow and also to quickly quantify the strains when used in subsequent trials.

### 2.3. Production of Acid and Gas from Carbohydrates

The pure cultures were inoculated in MRS broth added with 1% glucose, bromocresol green indicator (Sigma-Aldrich^®^, Buenos Aires, Argentina) 0.05 g/L and Durham bells [36] and incubated at 37 °C for 24 h. The colour change from green to yellow indicated the production of acid and the presence of gas manifested by the bubbles inside the bells were considered as positive reactions.

### 2.4. Lactic Acid Production

A modified method of Erkkilä et al. [7] was used. Strains were cultivated on MRS agar for 2 days at 37 °C. Three colonies of each strain were picked and inoculated in 10 mL MRS broth. After 48 h of incubation at 22 °C, the broths were purified with strong anion-exchange solid phase extraction cartridges (EFS-SPE). Lactic acid was separated with a reverse-phase column (LiChrosorb^®^ Hibar^®^ RP-18 250 × 4.6 mm, 5 µm) in a high-performance liquid chromatography (HPLC) system. The lactic acid concentration of the samples was determined at a wavelength of 210 nm by using the external standard method with 50, 100, 150 and 200 mM standards. The mobile phase (H_2_SO_4_ 50 mM) was pumped at 1.5 mL/min and the column temperature was 30 °C. The relative retention value for lactic acid was 4.8 min. Standards and each sample was run three times. The pH of the broths were measured at the beginning and after the incubation period. As a control the commercial starter *P. pentosaceus* PCFF-1 was used.

### 2.5. Effect of Temperature on the Growth of Probiotic LAB

LAB growth at different temperatures was observed in MRS broth after incubation at 7 and 15 °C for 14 days and at 22, 30, 37 and 43 °C for 7 days [37]. The first three values correspond to temperatures used commonly for the fermentation of raw-cured sausages; while 43 °C is the temperature used in the recovery tests for microorganisms from raw-cured sausages and is of interest for future studies. The cultures were inoculated at 1%. Optical density determinations at 600 nm (DO_600_) were performed with an automatic turbidimeter (Bioscreen C) and correlated with colony forming units (CFU) at the beginning (day 0) and after the incubation period.

### 2.6. Effect of pH, Sodium Nitrite and Sodium Chloride on Probiotic LAB Survival and Growth

To study in vitro the conditions of fermentation and maturation that occur during the preparation of a sausages and the combined effect of pH, sodium nitrite and sodium chloride on the strains, a modified method of Korkeala et al. [38] was used. The cultures were grown in 2 different media; fermentation medium with pH levels 4.5, 100 mg/L NaNO_2_ and 2% NaCl (*w*/*v*); and ripening medium with pH levels 5.5, 100 mg/L NaNO_2_ and 2% NaCl (*w*/*v*). Both media were prepared with MRS. MRS broth adjusted to pH 6.5 without NaNO_2_ and NaCl was used as a control. The wells of the microplates were seeded with 240 µL of medium and inoculated with 2.4 µL of 18 h culture. The determinations were made in triplicate. Growth was monitored using a Synergy™ HT (BioTek^®^, Winooski, VT, USA) Multi-Modal microplate reader. The microplates were incubated at 37 °C for 24 h and the optical density was measured every 30 min with a wide band filter (OD 620 nm). The delta absorbance (difference between the first and last absorbance reading), the gradient (slope of the logarithmic growth phase) and the lag phase (time when the bacterial growth started) were collected, analysed and exported with the Gen5 Data Analysis Software and finally used to characterise the growth. If no bacterial growth was observed the lag phase was assessed as 24 h.

### 2.7. Enzymatic Characterisation

The isolates were further characterised taking into account the following biochemical tests:

#### 2.7.1. Hydrogen Peroxide Production

The capacity of the strains to produce H_2_O_2_ was determined following the methodology described by McLean and Rosenstein [39]. Bacterial cultures were streaked on 15 mL of MRS agar (Britania^®^) supplemented with 2.5 mg/mL Tetramethylbenzidine (TMB) (Sigma-Aldrich^®^) and 0.01 mg/mL Peroxidase from radish (HRP) (Sigma-Aldrich^®^). The plates were incubated at 37 °C for 48 h under anaerobic conditions. After the incubation period, the plates were opened and exposed to atmospheric air for 30 min. After exposure, the colonies of the strains that produce H_2_O_2_ turned in blue. In the presence of H_2_O_2_ the HRP enzyme oxidises the TMB (colourless) to give rise to the formation of a blue pigment. When interpreting the results, it was considered that the hue of the blue colour was a semiquantitative measure of the amount of H_2_O_2_ produced and released into the medium by the strain [40]. From this, the hue of the blue colour was differentiated into the following categories: light blue (lower production) or dark blue (higher production). In the trial, *L. plantarum* Lp-UCC and *L. sakei* Ls-UCC strains were used as positive controls [41] and the commercial initiator *P. pentosaceus* PCFF-1 to evaluate its behaviour.

#### 2.7.2. Catalase Activity and Hydrogen Peroxide Hydrolysis 

To demonstrate the presence of the enzyme catalase (an enzyme capable of decomposing hydrogen peroxide in water and oxygen), the techniques described by Harrigan [42] were followed and tests were carried out on plates and tubes. The pure cultures were grown on MRS agar (Britania^®^), MRS broth (Britania^®^) and Nutritive broth (Britania^®^) at 37 °C for 24 to 48 h. After the incubation period, a few drops of 3% H_2_O_2_ were placed on the colonies grown in plates and 1 mL of the same solution was added to the cultures grown in tubes. The appearance of bubbles in the plates and in the broths caused by the release of oxygen, were considered positive tests. The tests were carried out in triplicate and *S. aureus* ATCC 25923 was used as a positive catalase control strain.

#### 2.7.3. Nitrate and Nitrite Reduction

The technique described in MacFaddin [43] was used to determine the ability of microorganisms to reduce nitrates to nitrites or free nitrogen gas. Nutritive broth (Britania^®^) with potassium nitrate (Sigma-Aldrich^®^) 1 g/L were made. Two reagents were used to reveal the presence of nitrites in the medium: reagent A (sulfanilic acid (Sigma-Aldrich^®^) at 0.8% in 5 N acetic acid) and reagent B (α-naphthylamine (Sigma-Aldrich^®^) 0.5% in acetic acid (Sigma-Aldrich^®^) 5 N). Nitrated broths were inoculated with pure cultures and incubated for 48 h at 37 °C. Assays were performed in triplicate and *S. aureus* ATCC 25923 and *S. xylosus* DD34 were used as control strains. After the incubation period 0.5 mL of reagent A was added and then 0.5 mL of reagent B. The appearance of red colour in the medium (after approximately 30 s) indicated the presence of nitrites in the medium. 

The reduction of nitrates can give other more reduced products such as ammonia, molecular nitrogen, nitric oxide, nitrous oxide or hydroxylamine, in this case, after the addition of the reagents, there is no colour change in the medium. To confirm that the process was negative, zinc powder (Sigma-Aldrich^®^) was added, which, if nitrates existed, reduced them to nitrites, turning the medium red and consequently indicating that previously nitrates had produced the reaction. If after the addition of zinc powder the colour did not turn red, this indicated that there was a reduction and that the product obtained was not nitrite but a smaller product.

#### 2.7.4. Milk Casein Hydrolysis

To examine protease activity, the methodology described by Harrigan [42] was followed with some modifications. Two culture media were prepared. (1) Skim milk agar: 5% skim milk powder and 1.3% agar (Britania^®^). (2) Skim milk agar with peptone: 2% skim milk powder in distilled water and agar (Britania^®^) at a concentration of 1.3% in peptone water (Britania^®^) were prepared separately; they were sterilised, allowed to cool to 45 °C and then homogenised and distributed in Petri dishes. Plates with 6 mm diameter perforations were prepared that were filled with 20 µL of fresh cultures and plates without perforations that were streaked with the same fresh cultures. *S. aureus* ATCC 25923 was used as a control. The plates were incubated at 37 °C for 48 to 72 h. The presence of a transparent halo around the grown colonies and wells was considered as a positive casein proteolysis reaction. The test was carried out in triplicate and on two different occasions.

#### 2.7.5. Lecithinase and Lipase Activity 

To examine the activity of microorganisms to produce the enzyme lecithinase and lipase, the methodology described in MacFaddin [43] was followed. Two culture media were prepared: Soy Tryptin agar (Britania^®^) and Brain Heart Infusion agar (Britania^®^) enriched with 10% sterile egg yolk. Plates with 6 mm diameter perforations were prepared which were filled with 20 µL of fresh cultures and plates without perforations were streaked with the same fresh cultures. *Staphylococcus aureus* ATCC 25923 was used as a positive control. The plates were incubated at 37 °C for 48 to 72 h. The hydrolysis of lecithin releases phosphorus and choline in stages, and the precipitation of insoluble fats (diglycerides) produces the opalescence of the medium. An opaque halo surrounding the colonies was considered as a positive lecithinase test, on the other hand, the enzyme lipase, catalyses the hydrolysis of triglycerides and diglycerides to fatty acids and glycerol, this, which is evidenced by an oily, iridescent shine on the colonies and around them, it was considered as positive lipase activity.

### 2.8. Antagonism against Staphylococcus Xylosus

To evaluate the antagonism of target microbes against *S. xylosus*, a sausage starter strain, a modified method of the agar spot test [44] and the agar diffusion technique [45] was used. Petri dishes were filled with MRS agar. A 2.6 µL amount of fresh culture of LAB strains was spotted onto and incubated at 37 °C for 24 h. The plates were overlaid with 15 mL of brain heart infusion (BHI)-1.5% agar (Britania^®^) inoculated with con 1.5 µL of fresh culture of *S. xylosus* and incubated at 37 °C for 24 h. The results were reported as positive or negative considering the presence or absence of clear areas of inhibition. In the agar diffusion technique, the production of inhibitory substances in cell-free extracts (CFE) of LAB strains was evaluated. LAB were grown in MRS broth for 24 h at 37 °C. They were then centrifuged at 5000× *g* in a 4 °C refrigerated centrifuge (IEC Multi RF Thermo, Spain) for 10 min. Subsequently, an aliquot of the supernatant was adjusted to pH 6.5 using NaOH as neutraliser to avoid acid inhibition (neutralised CFE). Another aliquot was used directly as a CFE without neutralising. Both of the supernatants were filter-sterilised, 0.22 µm pore diameter (Millipore, Merck, Germany). On a Petri dish with 15 mL of BHI solid agar (1.5% agar) were covered with 9 mL of semi-solid agar BHI (0.8% agar) inoculated with con 1.5 µL of fresh culture of *S. xylosus.* On the upper agar layer, various numbers of holes (6 mm) were punched out of the agar and were filled with 20 µL of neutralised and non-neutralised CFE of target microbes. The inoculated dishes were incubated at 4 °C for 1 h to allow diffusion of the extracts and then incubated at 37 °C for 24 h and examined for clear inhibition halos around the wells. The results were reported as positive or negative considering the presence or absence of inhibition zones, respectively.

### 2.9. Amino Acid Decarboxylation

To evaluate the amino decarboxylase activity [46] a qualitative method was used. A culture medium was prepared with the following composition: Casein peptone (Sigma-Aldrich^®^) 0.5%, Yeast extract (Oxoid^®^, Basingstoke, United Kingdom) 0.3%, D (+) Glucose (Biopack^®^,) 0.1% and bromocresol purple (Sigma-Aldrich^®^) 0.0016%. Then, 0.5% of the amino acid to be tested was added to 0.9% of the base medium, in this case L-Arginine (Anedra^®^, Buenos Aires, Argentina), L-Histidine (Anedra^®^), L-Lysine (Anedra^®^), L-Tyrosine (Anedra^®^) and L-Tryptophan (Anedra^®^). The pH of the medium was then adjusted to 6.7 ± 0.1 at 25 °C. The tubes were inoculated with 10 µL of 24 h culture and incubated 72 h at 37 °C in under aerobic conditions. The colour of the medium at the time of inoculation was purple. In addition to the probiotic strains, the *P. pentosaceus* PCFF-1, *L. plantarum* Lp-UCC and *L. sakei* Ls-UCC strains were used in the assay to observe how they behaved with respect to the amino acids tested. In cases where the strain did not decarboxylate the amino acid, the colour of the medium remained yellow until the end of the test and the test was considered negative. On the contrary, when the strain decarboxylated the amino acid the colour turned purple and the test was considered positive.

### 2.10. Antagonism against Pathogens Associated with Sausages

The antagonism of the probiotic strains against the pathogens *E. coli*, *L. monocytogenes*, *Salmonella* Dublin and *S. aureus* was studied according to the method of the agar spot test described by Lewus et al. [44], the agar diffusion technique described by Tagg and McGiven [45] indicated above and the microplate antimicrobial activity described by Ruíz-Moyano et al. [47] with some modifications.

In the antimicrobial activity in microplates, inhibitory effects of strains on selected potential harmful microorganisms were studied by following the ability of the target microbes to grow in a medium containing sterilised neutralised supernatants from probiotic strain cultures. The ability of fresh selected harmful microorganisms to grow in broth (BHI) supplemented with of filter-sterilised supernatant was evaluated by following the microbial growth at 37 °C for 24 h with an automated turbidimeter Synergy™ HT (BioTek^®^) Multi-Modal microplate reader. Each well of the microplates was seeded with 120 µL of double concentration BHI broth supplemented with 120 µL of neutralised CFE of the probiotic strain under study. The wells were inoculated with 2.4 µL of 18 h culture of target pathogen. Simple concentration BHI broth, pH 6.5 without supplementation, inoculated with each of the pathogenic strains, was used as a positive control. The sowing was done in triplicate. The optical density was measured with a wide band filter (OD 620 nm) every 30 min. The delta absorbance (difference between the first and last absorbance reading), the gradient (slope of the logarithmic growth phase) and the lag phase (time when the bacterial growth started) were collected, analysed and exported with the Gen5 Data Analysis Software (BioTek^®^) and finally used to characterise the growth. If no bacterial growth was observed the lag phase was assessed as 24 h. The inhibitory effect of probiotic culture supernatants was determined by comparing the maximum growth with respect to the positive control.

### 2.11. Statistical Analysis

To quantify the strains, a logarithmic regression model was used. The model was built with OD600 data and the decimal logarithm of microbial growth at 37 °C [48]. Lactic acid production data, growth in fermentation and maturation conditions and antimicrobial activity represented by continuous variables were analysed using an ANOVA. All the experiments in the present study were conducted in triplicate and the values presented in results are mean values from each triplicate. The experimental data obtained after mean from each triplicate set was statistically evaluated at a significance level of *p* ≤ 0.05. The IBM SPSS Statistics 19 program was used. When the differences were significant (*p* ≤ 0.05), the Duncan multiple means comparisons test was applied.

## 3. Results

The strains studied showed different technological capabilities, compared to the different conditions in which the tests were performed. These results are presented in Table 2, Table 3 and Table 4 and Table S1. To be able to select the most suitable strains, we performed a ranking with a numerical categorisation, assigning positive and negative values according to the results obtained for the different parameters measured (Table 5).

In general, they showed very good capacity to generate biomass in a short period of time (37 °C; 12 h; Table 2). All reached more than 8 log_10_ CFU/mL in the time tested. The logarithmic model used adjusted significantly (*p* ≤ 0.01) and allowed quantifying the strains studied (Figure S1). The strains with the highest number of cells per unit volume were *L. casei* Shirota, *E. faecium* MXVK29, *L. rhamnosus* R0011 and *L. rhamnosus* Lr-32.

All tested strains fermented glucose, producing lactic acid without gas generation, indicating that their metabolism is homofermentative (Table 2).

The amount of lactic acid produced by each strain is shown in Table 2. The highest concentration was generated by the commercial initiator *P. pentosaceus* PCFF-1 (*p* ≤ 0.05), while *L. reuteri* DSM 17918, *L. reuteri* DSM 17938 and *L. helveticus* R0052 had the lowest production. This was related to their low capacity to grow at the incubation temperature of the trials (22 °C), which was chosen because it is the usual fermentation temperature of sausages in which this strain is intended to be used in the future. The strains *L. rhamnosus* R0011, *L. rhamnosus* Lr-32, *L. paracasei* Lpc-37, *L. casei* Shirota and *E. faecium* MXVK29 generated lactic acid in the range of 216 to 148 mM (Table 2).

The results in Table 2 show that at 7 °C *L. helveticus* R0052, *L. reuteri* DSM17918 and *L. reuteri* DSM17938 did not increase their biomass; *L. rhamnosus* Lr-32 had slow development but the strain remained viable and an increase was observed on day 15 of the incubation period. Between 30 and 37 °C, the most characteristic temperature of the fermentation of central European meat products, all strains had good growth, which indicates that they are mesophilic and that it corresponds to their optimum growth temperature. At 43 °C the strains *L. rhamnosus* R0011, *L. rhamnosus* Lr-32, *E. faecium* MXVK29, *L. helveticus* R0052, *L. reuteri* DSM17918 and *L. reuteri* DSM17938 had good growth. The first three of the above strains showed a wide optimum range of growth temperatures. For *L. helveticus* R0052, *L. reuteri* DSM17918 and *L. reuteri* DSM17938 the range was narrower and established only at the upper levels. *L. paracasei* Lpc-37 and *L. casei* Shirota did not grow at high temperature (Table 2).

The strains *L. rhamnosus* Lr-32, *L. reuteri* DSM17918 and *L. reuteri* DSM17938 were best adapted to the simulated fermentation conditions (pH 4.5, NaCl 2% and NaNO_2_ 100 mg/L). The Nmax values of these strains were similar and, in turn, higher and different from the rest of the strains studied (*p* ≤ 0.05). On the contrary, it was observed that *E. faecium* MXVK29 failed to develop under the conditions studied (Table 2). On the other hand, in the ripening stage (pH 5.5, 3% NaCl, 200 mg/L NaNO_2_) it can be seen that *L. rhamnosus* Lr-32 and *L. rhamnosus* R0011, *L. paracasei* Lpc-37, *L. reuteri* DSM17918 and *L. reuteri* DSM17938 had the highest Nmax values (*p* ≤ 0.05) (Table 2).

The strains that produce H_2_O_2_ showed that their white colonies turn to light blue colour (in the case of *P. pentosaceus* PCFF-1, *L. plantarum* Lp-UCC, *L. sakei* Ls-UCC and *L. casei* Shirota) and intense blue, indicating a high production of hydrogen peroxide, (for *L. helveticus* R0052, *L. reuteri* DSM17918 and *L. reuteri* DSM17938) (Table 2). All strains were negative for catalase activity and were not observed to breakdown H_2_O_2_. Nor were any of the tested trains found to reduce nitrates (Table 2).

The probiotics strains studied showed no inhibition halos against *S. xylosus* DD-34 (a CNC strain commonly used in the meat industry) (Table 2). 

Probiotics *L. rhamnosus* R0011, *L. rhamnosus* Lr-32, *L. paracasei* Lpc-37, *L. casei* Shirota and *E. faecium* MXVK29 hydrolysed milk casein generating a transparent halo around the colonies. These strains also hydrolysed triglycerides, showing an oily shine on the colonies and around them (Table 2). 

*Lactobacillus rhamnosus* R0011, *L. rhamnosus* Lr-32 and *L. paracasei* Lpc-37 strains did not decarboxylate any of the amino acids tested. However, the other tested strain decarboxylated at least one amino acid after 24 or 36 h (Table S1).

Table 3 shows the results of the agar spot test. Here it can be seen that all probiotic strains tested generated halos of inhibition against the pathogens *L. monocytogenes* and *S. aureus*. Except for *L. helveticus* R0052, probiotics formed inhibition halos against *E. coli* and *Salmonella* Dublin. In the agar diffusion technique, only the *E. faecium* MXVK29 strain was able to inhibit *L. monocytogenes* in both non-neutralised and neutralised CFE (Figure S2).

Table 4 shows the results of microbial antagonism in microplate. The CFE of the different probiotics produced lower growth of *E. coli*, *Salmonella* Dublin and *S. aureus* (*p* ≤ 0.05). The *L. casei* Shirota strain had the greatest antagonistic effect against *S. aureus*. The *L. paracasei* Lpc-37, *E. faecium* MXVK29 and *L. reuteri* DSM17918 strains strongly inhibited the growth of *Salmonella* Dublin while the *E. faecium* MXVK29 and *L. reuteri* DSM17938 were the strains with the bigger inhibition against *E. coli*. There was no significant difference (*p* ≤ 0.05) in the growth of *L. monocytogenes* in the control medium with respect to media containing CFE of *L. rhamnosus* R0011 and *L. helveticus* R0052. On the contrary, there were, with the CFE of the other probiotics tested and a pronounced inhibition effect was evidenced in the medium containing CFE of the *L. reuteri* DSM17938 strain.

Table 5 shows the results of a strain selection ranking. There it emerged that the most suitable strains to be selected as starter are *L. rhamnosus* Lr-32, *L. rhamnosus* R0011, *L. paracasei* Lpc-37, *E. Faecium* MXVK29 and *L. casei* Shirota. Of the aforementioned strains, the one that best tolerated the fermentation and maturation conditions of sausages was *L. rhamnosus* Lr-32.

## 4. Discussion

The growth shown by the strains studied, all above 8 log_10_ CFU/mL in 12 h was important. This characteristic is related to the adaptive power of microorganisms to the environment and its ability to develop rapidly and is essential when producing probiotic products as they should be able to deliver an adequate amount of live organisms that enable the health-promoting effects [11]. Further, when microorganisms are incorporated into sausage batter, they must grow rapidly and reach high numbers to become dominant and competitive against the indigenous microbiota present in raw materials [18,19].

For the fermentation process to occur, the participation of LAB is necessary [15]. The ability of the strains studied to produce only lactic acid from carbohydrates was very important. Heterofermentative LAB are not suitable for sausage production due to the formation of carbon dioxide, which accumulates in the matrix and generates holes of different sizes in the product [26]. In addition, these LAB produce concentrations of acetic acid that cause a strong off flavour. 

The aptitude of starter strains to fast acidification is an important characteristic as it has an impact on taste, safety, aroma and bacteriostatic or bactericidal properties [49,50]. The main function of LAB is to reduce the pH of the matrix through production of lactic acid from the fermentation of carbohydrates. The production of acids in dry sausage depends on the type and concentration of sugars added to the meat mixture, the diameter of the dry sausage and the LAB microbiota [11]. A reduction in pH is necessary for fibrillar proteins to coagulate, resulting in improved firmness and cohesiveness of the final product, facilitating slicing [51,52]. They also promote the spontaneous reduction of nitrites to nitric oxide, which reacts with myoglobin to form nitrosomyoglobin, the compound responsible for the typical red colour of cured sausages [53]. In addition, they contribute to the flavour of the final product through the formation of its typical acidic taste. The acidity reached by the meat matrix also contributes to increasing the activity of cathepsin D, which is responsible for muscle proteolysis [54]. The production of organic acids is undoubtedly the determining factor on which the product’s shelf life and safety depends. The inhibition of pathogens and spoilage microbiota also depends on a rapid and adequate production of organic acids and the associated reduction in pH [15]. Finally, it has been reported that the rapid decrease in pH caused by negative aminodecarboxylase cultures decreases the production of biogenic amines in sausage [55]. The immediate and rapid formation of acid at the beginning of the fermentation process, and the production of sufficient quantity of organic acids that allow to reach a pH below 5.1 is an essential requirement of the starter LAB. However, excessive acid formation is often associated with colour defects (due to inhibition of CNCs) and sometimes with gas formation (in the case of heterofermentative bacteria), one of the most important problems in sausage processing [26]. Comparing the results of this work with those obtained by Erkkilä et al. [7] it can be assumed that the studied strains produced enough lactic acid to act as the main initiating microorganism in sausage production. However, a sausage test should be performed to confirm this hypothesis. The acid taste of fermented meat products, which is correlated to the acid content, is appreciated in some countries whereas it is undesirable in others [11]. Therefore, the acid production is an important factor for selection of strains as starter cultures in fermented sausages also considering the characteristics of the product to be produced.

The manufacturing temperature of fermented meat sausages ranges between 4 and 7 °C when the mixture is prepared, rises between 18 and 24 °C during the fermentation period and is reduced from 12 to 15 °C during the drying and ripening period [21,56]. Therefore, it is necessary to select isolates of LAB which are capable of developing over a wide range of temperatures [15]. In the temperature range of the typical Mediterranean fermentation (15–22 °C), which is also generally used in the Argentinean meat industry, the strains *L. rhamnosus* R0011, *L. rhamnosus* Lr-32, *L. paracasei* Lpc-37, *L. casei* Shirota and *E. faecium* MXVK29 were the ones that best adapted and developed. 

The growth of the LAB strains under the conditions of fermentation and maturation of the sausages is decisive so that they can be considered as a potential starter. The strains studied showed a different adaptive response to the fermentation and drying conditions studied. Three of the strains studied (*L. reuteri* DSM 17938, *L. reuteri* DSM 17918 and *L. rhamnosus* Lr-32) showed the best performance in both conditions. The concentration of salt added to raw-cured sausage batter, approximately 2% depending on each product, can reach up to 2.5–3.5 *w*/*w* in the final product [57]. The initial pH of the mixture, which is generally around 6, decreases during fermentation and reaches values between 4.6–5.1. Subsequently, yeasts can increase the pH of the product, reaching final values, ranging from 5.1–5.5 [21,57]. Bacterial growth in food matrices may be affected by intrinsic and extrinsic factors. Therefore, it is necessary to select isolates of LAB which are capable of developing tolerating adverse conditions including the presence of sodium chloride, sodium nitrite and acidic pH, it is essential that they adapt properly to the conditions of the food matrix. Thus, the above strains will present a greater capacity to persist during the fermentation of food [15]. The development of a new probiotic fermented meat product requires the application of probiotic bacteria that are resistant to salt, nitrite and acidic pH, so that they are able to activate and grow rapidly during fermentation and maturation [16]. This will allow competition with the natural microbiota and result in a successful fermentation. Further, when producing functional foods with probiotic cultures, it is necessary that the product contains a high enough number of viable cells at the time of consumption, which must exceed the minimum suggested dose for a health benefit. This is a prerequisite to exert beneficial effects on the host [57]. Tolerance to high salt concentration and fast acidification constitute key functions for a significant role of the organisms in meat fermentation [11].

Most lactobacilli are capable of forming hydrogen peroxide through the enzyme lactate oxidase during sausage fermentation. Hydrogen peroxide can interfere with the organoleptic properties of fermented meat products by increasing the rancidity due to lipid oxidation [15] and discolouration of the final product [21]. Authors’ trials have reported that *L. plantarum* and *L. sakei* are capable of producing H_2_O_2_, which is an oxidising agent, and it has been suggested that such strains could be related to defects in colour and flavour of the sausage [16,41]. Taking into account the aforementioned, *L. helveticus* R0052, *L. casei* Shirota, *L. reuteri* DSM17918 and *L. reuteri* DSM17938 would not be the most suitable strains to be used as sausage starters. However, it should also be considered that the LAB strains will be part of a mixed starter culture and that the CNCs are capable of lysing the H_2_O_2_ produced [15].

Some LAB strains involved in the fermentation of meat such as *L. sakei*, *L. plantarum*, *L. pentosus* and *P. acidilactici* have haemo-dependent catalase activity that is active in meat products, since these substrates contain abundant hemin [21]. Although all the probiotic strains studied were catalase negative and its activity of LAB used as starter cultures in meats, it is a desirable property [21], it is not essential since this function is performed by the CNCs [15] that are part of the starter culture. CNCs can neutralise pro-oxidant molecules, limiting the oxidative processes based on their superoxide dismutase (SOD) and catalase (CAT) [15].

Although several authors have reported that some meat LABs have nitrate reductases [21,22,58] the probiotic strains tested did not reduce nitrates but Gram-positive cocci species contribute to the formation of the typical characteristics of fermented meat products, such as colour. Nitrate reductase confers a characteristic colour to the product [15]. The probiotic LAB will be part of a mixed culture along with a micrococcus.

Starter cultures affect the aroma and taste of fermented meat products and the use of their enzymatic pattern as selection criterion could be of interest [16]. Five of the strains studied showed their lipolytic and proteolytic capacity, which is interesting as a technological property. This is a desirable characteristic, which could contribute to the catabolism of proteins and fats by generating precursors of sausage flavour compounds [21]. These activities are associated with flavour development [59,60].

As mentioned above, the starter cultures of meat products are mainly a mixture of LAB and CNC strains, and it has been used as a commercial starter for many years for sausage making in different countries worldwide [11]. To carry out the expected functions it is necessary that the starter LAB strain show tolerance or even synergism with the CNC that is part of starter culture [21]. Therefore, the results obtained indicate that the probiotic strains studied could be combined with *S. xylosus* DD-34 thus forming a mixed starter culture to be used in the preparation of probiotic sausage. It has been shown that some of these coagulase negative staphylococci could affect both growth and proteolytic activities of LAB strains. Such interactions are therefore of interest and should be considered for selection of starters in order to improve the organoleptic properties of fermented sausages [61].

Biogenic amines (BA) are organic bases of low molecular weight, polar or semi-polar compounds, resulting from the decarboxylation of amino acids [62]. These compounds are usually non-toxic, but when ingested in high amounts by people with gastrointestinal diseases, or incapable of detoxification due to genetic problems and/or combined with the ingestion of alcohol, they can cause nausea, diarrheal and hyperdilation of blood vessels [62]. The most prevalent BAs in food and beverages are histamine, tyramine, putrescine, cadaverine and β-phenylethylamine [62]. Residual nitrite present in fermented sausages can react with amines and form carcinogenic nitrosamines [63]. The results indicate a low capacity of the microorganisms studied to generate biogenic amines, especially the probiotic strains, which did not decarboxylate any of the amino acids tested. Previous studies have reported deamination of arginine by strains of *L. sakei* but not by strains of *L. plantarum* [16,64]. Other authors reported the formation of biogenic amines by both *L. plantarum* and *L. sakei* strains [47]. The absence of biogenic amines formation has been proposed as a selection criterion for new strains used as starter cultures for meats [21,26,47]. The rapid growth and production of acid will further prevent the development of an indigenous microbiota that produces amines [21]. In addition, other studies indicate that the use of amino decarboxylase-negative starter cultures significantly reduces the levels of biogenic amines formed in sausage [8,65]. This activity has already been described in some LABs for fermentation of sausage, such as *L. plantarum* and *L. casei* [66]. The most promising microorganisms utilised as starter cultures are identified microorganisms, previously characterised as safe and exhibiting desired metabolic activity [67]. Based on these concepts, the probiotics studied *L. rhamnosus* R0011, *L. rhamnosus* Lr-32 and *L. paracasei* Lpc-37 would be the main candidates to be used as a starter culture in the preparation of a healthy and safe sausage.

LAB are known to promote food safety and quality because they present antagonistic activity against spoilage and pathogenic microorganisms [68]. In addition to the use as a starter microorganism, based mainly on acidification capacities, the antibacterial capacity demonstrated by the studied strains could help to develop biopreservation properties in dry fermented meat products. These approaches appear promising for future development of biopreservation for enhanced shelf life and safety of meat products [50]. Antagonistic activity may occur through several mechanisms, including competition for nutrients and adhesion sites, as well as production of bactericidal compounds such as organic acids (lactic, acetic and propionic), carbon dioxide, diacetyl, hydrogen peroxide, reuterin and bacteriocins [68]. The main antimicrobial effect of LAB responsible for biopreservation of foods is their acidification capacity. However, bacteriocins produced by LAB can provide additional control against pathogens in meat sausage [15]. The use of a starter culture capable of reducing or inhibiting the growth of pathogenic microorganisms associated with sausage would contribute to ensuring product safety. The ability to inhibit pathogens capable of producing foodborne illnesses is a quality of interest for the starters used in the manufacture of raw-cured meat products [26]. Although this effect is usually restricted to a limited number of microorganisms, a broad inhibitory effect is expected in strains already characterised as those used in this work. Further on this characteristic, focus was on pathogen inactivation, as probiotic strains with additional food safety properties could confer added value to healthy fermented meat products.

According to the data obtained, *L. rhamnosus* Lr-32, *L. rhamnosus* R0011, *L. paracasei* Lpc-37, *E. faecium* MXVK29 and *L. casei* Shirota strains are the main candidates to be used as sausages starters. These results coincide with those obtained by Rebucci et al. [69] who proposed the strains of *L. casei* and *L. rhamnosus* isolated from sausages are the best potential functional starter cultures in meat products. Accordingly, recent investigations have shown that strains of *L. rhamnosus*, *L. fermentum* and *L. paracasei* of human intestinal origin were able to survive the dry sausage manufacturing process, being detected in high numbers in the final product [7,8,9,10,11,12,14,70].

Considering that the adaptability and growth of the LAB strains under the fermentation and maturation conditions of the raw-cured sausages is a decisive selection criterion, *L. rhamnosus* Lr-32 is considered the best of the strains studied to be tested as an initiator in the preparation of such sausages. A probiotic starter culture adapted to the ecology of the meat fermentation, with those potential properties and antimicrobial activity could provide significant health benefits and contribute to enhance the hygienic quality of the products [16]. We emphasised that strain *L. rhamnosus* Lr-32 is of interest for further tests in sausages according to the results obtained. Although the strain had excellent results in vitro, its performance as an initiator in sausages will be dependent on its ability to survive and fulfil its functions reliant on the raw materials, the conditions of preparation of the sausages and the existing indigenous microbiota communities.

## 5. Conclusions

The objective of this study was to evaluate technological and safety properties of eight commercial probiotic strains to select those that were most suitable to act as a sausages starter microorganism. In addition to contributing to human health, probiotic fermented meats need to be of sufficient commercial value. According to the data obtained *L. rhamnosus* Lr-32, *L. rhamnosus* R0011, *L. paracasei* Lpc-37, *E. faecium* MXVK29 and *L. casei* Shirota strains are the main candidates to be used as sausages starters because they showed very good capacity to grow, a high production of lactic acid without gas formation, generate good growth at the low temperatures at which this product is manufactured, demonstrated to have proteolytic and lipolytic capacity that could contribute to flavour and did not show antagonism against the CNC, that are usually part of the starter culture mix. These strains did not exhibit amino acid decarboxylase activity, a key requirement for the selection of a new safe LAB starter culture. In addition, they could prevent the growth of the native biogenic amine-producing microbiota, helping to produce a safer product. The strains showed antimicrobial activity against harmful microorganisms frequently associated with sausage, which could contribute to improving the hygienic quality of the product and preventing possible diseases. 

The theoretical basis for the selection of a new starter culture includes technological aspects (growth in food base, sensory properties, stability and viability) and safety as mentioned above. The beneficial effects of LAB have been attributed to their ability to suppress the growth of pathogens, probably by secretion of antibacterial substances such as lactic acid and bacteriocins. Of the aforementioned strains, *L. rhamnosus* Lr-32 was the strain that best tolerated the levels of salt, nitrate and the low pH during the simulated stages of fermentation and maturation of sausage.

The novelty of this work was to study existing probiotics strains as starter and to show that the probiotic strain could be used without being combined with a commercial starter culture. It is expected that the strain fulfils both functions being a starter at the same time as a beneficial microorganism.

The great advance that could be achieved in the science of the meat using probiotic microorganism as a starter would be a functional meat product with improvements in the safety aspect of the product, potential benefits to the health of the consumer and it could improve the negative image that some consumers have about sausages.

It is necessary to carry out new studies in manufacturing conditions of the raw-cured sausages in order to verify if probiotic *L. rhamnosus* Lr-32 maintains the characteristics identified in the in vitro work presented here.

## Figures and Tables

**Table 1:** Bacterial strains used in the tests.

Species	Strain	Manufacturer/Origin
**Probiotics**		
*Lactobacillus rhamnosus*	R0011	Lallemand
*Lactobacillus helveticus*	R0052	Lallemand
*Lactobacillus rhamnosus*	Lr-32	DuPont
*Lactobacillus paracasei*	Lpc-37	DuPont
*Lactobacillus casei*	Shirota	Yakult
*Enterococcus faecium*	MXVK29	Autonomous Metropolitan University, México, Mexico
*Lactobacillus reuteri*	DSM17918	BioGaia
*Lactobacillus reuteri*	DSM17938	BioGaia
**Sausage starters**		
*Lactobacillus plantarum*	Lp-UCC	Catholic University of Cordoba, Cordoba, Argentina
*Lactobacillus sakei*	Ls-UCC	Catholic University of Cordoba, Cordoba, Argentina
*Pedicoccus pentosaceus*	PCFF-1	Chr. Hansen
*Staphylococcus xylosus*	DD-34	Chr. Hansen
**Sausage associated pathogens**		
*Listeria monocytogenes*		Pediatric Hospital of the Infant Jesus, Cordoba, Argentina
*Staphylococcus aureus*	ATCC 25923	Pediatric Hospital of the Infant Jesus, Cordoba, Argentina
*Escherichia coli*	ATCC 25922	DSPV-FCV-UNL, Esperanza, Argentina
*Salmonella Dublin*	DSPV 595T	DSPV-FCV-UNL, Esperanza, Argentina

**Table 2:** Technological and safety features of commercial probiotic bacteria.

	*L. rhamnosus* R0011	*L. helveticus* R0052	*L. rhamnosus* Lr-32	*L. paracasei* Lpc-37	*L. casei* Shirota	*E. faecium* MXVK29	*L. reuteri* DSM17918	*L. reuteri* DSM17938
Biomass generation (log_10_ CFU/mL)								
	9.4 ± 0.08	8.3 ± 0.10	9.3 ± 0.13	8.6 ± 0.07	9.7 ± 0.10	9.6 ± 0.10	9.2 ± 0.10	9.3 ± 0.08
Growth at (log_10_ CFU/mL)								
7 °C	8.9 ± 0.13	ng	8.3 ± 0.10	8.5 ± 0.11	8.8 ± 0.19	9.4 ± 0.36	ng	ng
15 °C	9.3 ± 0.18	ng	9 ± 0.10	8.6 ± 0.37	9.6 ± 0.33	9.5 ± 0.31	ng	ng
22 °C	9.3 ± 033	7.5 ± 0.32	9.1 ± 0.16	8.6 ± 0.24	9.7 ± 0.10	9.6 ± 0.80	ng	ng
30 °C	9.4 ± 0.40	8.3 ± 0.25	9.3 ± 0.35	8.6 ± 0.36	9.7 ± 0.22	9.6 ± 0.13	9.2 ± 0.40	9.3 ± 0.14
37 °C	9.4 ± 0.50	8.3 ± 0.30	9.3 ± 0.33	8.6 ± 0.33	9.7 ± 0.50	9.6 ± 0.51	9.2 ± 0.45	9.3 ± 0.07
43 °C	9.2 ± 0.20	8.3 ± 0.53	9.3 ± 0.25	ng	ng	9.5 ± 0.09	9.2 ± 0.40	9.3 ± 0.35
Gas production								
	nd	nd	nd	nd	nd	nd	nd	nd
Acid production at (nmol/L) *								
22 °C	215.94 ^A^ ± 0.52	28.27 ^D^ ± 0.39	177.44 ^B^ ± 0.15	198.29 ^A^ ± 0.42	201.52 ^A^ ± 0.59	147.85 ^C^ ± 0.91	7.5 ^E^ ± 0.41	7.06 ^E^ ± 0.54
Growth (OD620) simulated sausages conditions *								
Fermentation	0.57 ^D,E^ ± 0.06	0.77 ^B,C,D^ ± 0.06	0.90 ^A,B,C^ ± 0.17	0.63 ^C,D^ ± 0.12	0.30 ^E,F^ ± 0.17	0.00 ^F^ ± 0.00	1.07 ^A,B^ ± 0.04	1.17 ^A^ ± 0.06
Ripening	1.10 ^A,B^ ± 0.17	0.60 ^C,D^ ± 0.10	1.27 ^A,B^ ± 0.12	1.23 ^A,B^ ± 0.23	0.93 ^B,C^ ± 0.15	0.33 ^D^ ± 0.29	1.33 ^A^ ± 0.12	1.07 ^A,B^ ± 0.32
Catalase activity								
	nd	nd	nd	nd	nd	nd	nd	nd
H_2_O_2_ production								
	nd	d	nd	nd	d	nd	d	d
Nitroreductase activity								
	nd	nd	nd	nd	nd	nd	nd	nd
Proteolytic activity								
	d	nd	d	d	d	d	nd	nd
Lipolytic activity								
	d	nd	d	d	d	d	nd	nd
Antagonism against *S. xylosus*; coagulase negative cocci (CNC)								
	nd	nd	nd	nd	nd	nd	nd	nd
Antagonism against pathogens								
*S. aureus*	d	d	d	d	d	d	d	d
*L. monocytogenes*	nd	nd	d	d	d	d	d	d
*S. dublin*	d	d	d	d	d	d	d	d
*E. coli*	d	d	d	d	d	d	d	d

(ng): no growth; (d): detected; (nd): not detected; (*, ^A–F^): Different letters indicate significant differences (*p* ≤ 0.05).

**Table 3:** Antimicrobial activity of probiotic strains against pathogens associated with fermented sausages.

	Inhibitory Strain															
	Colony Diameter (mm)								Clearing Zone Diameter (mm)							
Target Strain	R0011	R0052	Lr-32	Lpc-37	Shirota	MXVK29	DSM17918	DSM17938	R0011	R0052	Lr-32	Lpc-37	Shirota	MXVK29	DSM17918	DSM17938
*S. aureus*	7	6.5	8	7.5	7	6.5	8	7.5	14	4	12	14	9	7	10	11
*L. monocytogenes*	7	6	9	8	7	7	9	8	15	5	12	15	13	15	7	8
*Salmonella* Dublin	5	5	7	7	7	5	7	7	12	nd	16	14	16	12	11	10
*E. coli*	5	5	6.5	7	6	6	7	6.5	12	nd	12	17	11	8	8	10

**Table 4:** Antimicrobial activity of probiotic strains against pathogens associated with fermented sausages.

Target Strain *	Growth (OD_620_)								
	Control	R0011	R0052	Lr-32	Lpc-37	Shirota	MXVK29	DSM17918	DSM17938
*S. aureus*	1.13 ^C^	1.0 ^B^	1.03 ^B^	1.0 ^B^	1.0 ^B^	0.9 ^A^	1.0 ^B^	1.03 ^B^	1.0 ^B^
*L. monocytogenes*	1.03 ^D^	1.0 ^D^	1.03 ^D^	0.90 ^C,D^	0.70 ^B^	0.80 ^B,C^	0.70 ^B^	0.80 ^B,C^	0.40 ^A^
*S.* Dublin	1.40 ^E^	1.20 ^D^	1.20 ^D^	1.10 ^C^	0.97 ^A,B^	1.10 ^C^	0.90 ^A^	0.90 ^A^	1.0 ^B^
*E. coli*	1.57 ^E^	1.27 ^D^	1.23 ^D^	1.10 ^C^	1.10 ^C^	1.10 ^C^	0.93 ^A^	1.03 ^B,C^	1.0 ^A,B^

(*, ^A–E^): Different letters indicate significant (*p* ≤ 0.05) differences.

**Table 5:** Strain selection criteria according to results obtained.

	*L. rhamnosus* Lr-32	*L. rhamnosus* R0011	*L. paracasei* Lpc-37	*E. faecium* MXVK29	*L. casei* Shirota	*L. reuteri* DSM17918	*L. reuteri* DSM17938	*L. helveticus* R0052
Biomass generation	1	1	1	1	1	1	1	1
Growth at all different temperatures tested	1	1	0	1	0	0	0	0
No gas production	1	1	1	1	1	1	1	1
Acid production	1	1	1	1	1	0	0	0
Growth at fermentation condition	1	0	0	0	0	1	1	0
Growth at ripening condition	1	1	1	0	0	1	1	0
Catalase activity	0	0	0	0	0	0	0	0
No H_2_O_2_ production	1	1	1	1	0	0	0	0
Nitroreductase activity	0	0	0	0	0	0	0	0
Proteolytic activity	1	1	1	1	1	0	0	0
Lipolytic activity	1	1	1	1	1	0	0	0
Antagonism against *S. xylosus*	1	1	1	1	1	1	1	1
Antagonism against all pathogens	1	0	1	1	1	1	1	0
No amino acid decarboxylation	1	1	1	0	0	0	0	0
Total number of positive reaction results	12	10	10	9	7	6	6	3

(0): negative result to the desired reaction; (1): positive result to the desired reaction.

## Supplementary Materials

The following are available online at https://www.mdpi.com/2304-8158/9/5/596/s1, Figure S1: Standard calibration curves used to quantify probiotic LAB; Figure S2. Inhibition of *Listeria monocytogenes* by *Enterococcus faecium* MXVK29 in agar well diffusion assay; Table S1. Amino acid decarboxylation assay.
