# Supplementary material for: Technological Characterisation of Probiotic Lactic Acid Bacteria as Starter Cultures for Dry Fermented Sausages
Source: Foods. 2020 May 7;9(5):596. doi: 10.3390/foods9050596 (PMC7278822; doi:10.3390/foods9050596)
Supplement: Supplementary file 1 [file foods-09-00596-s001.docx]

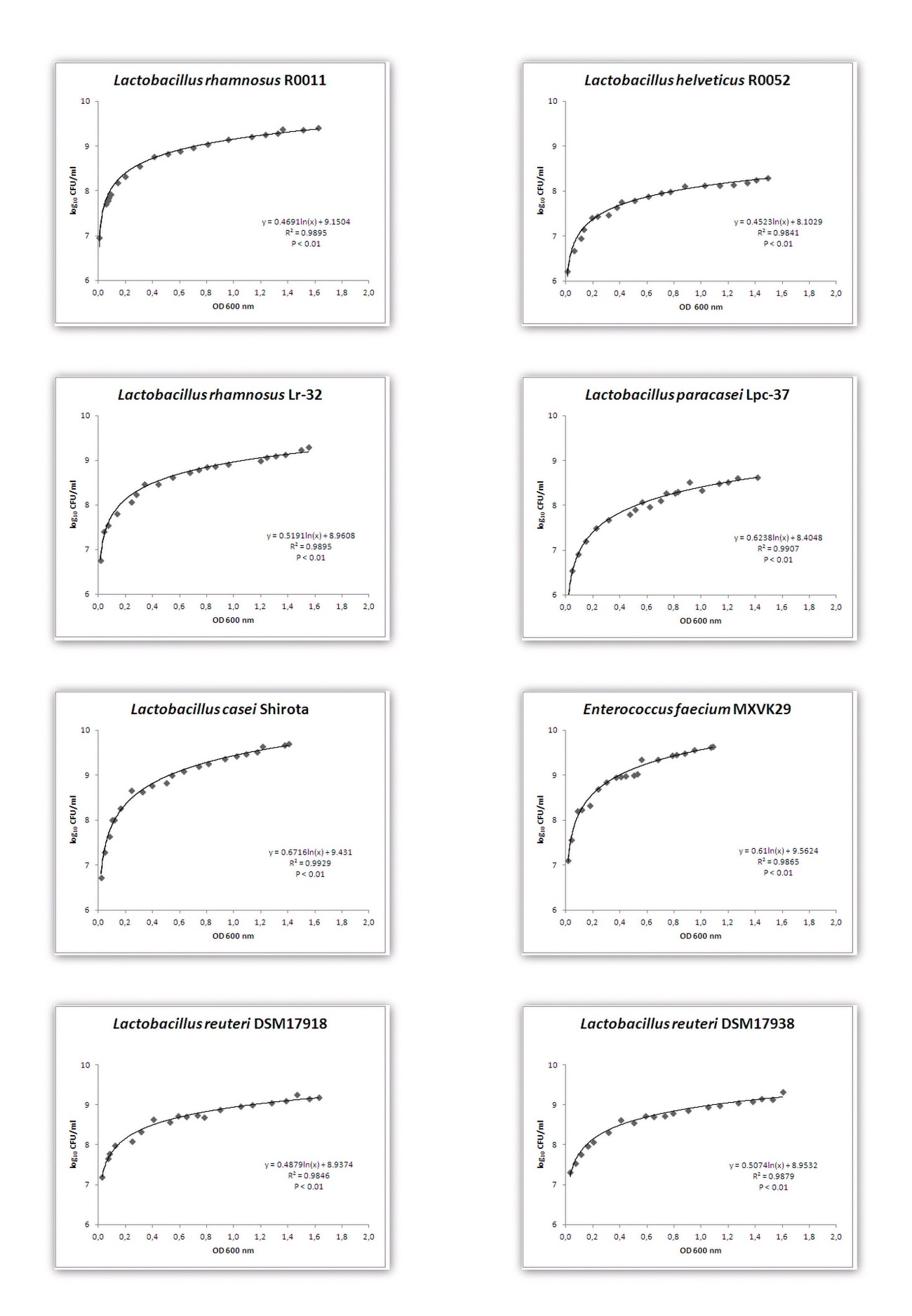


Figure S1. Standard calibration curves used to quantify probiotic BAL.

**
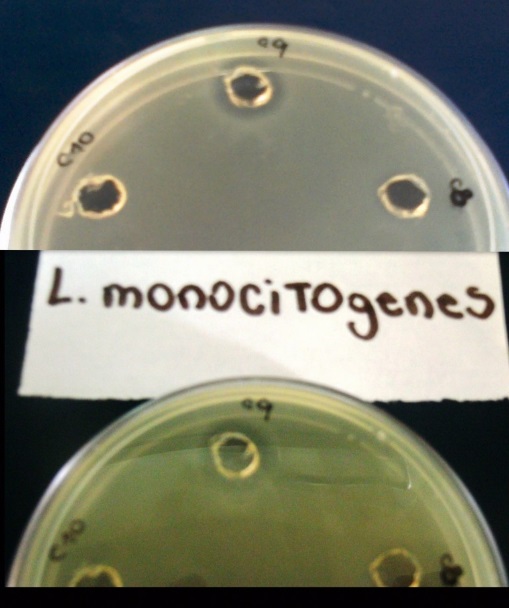
**

Figure S2. Inhibition of *Listeria monocytogenes* by *Enterococcus faecium* MXVK29 in agar well diffusion assay.

**Table S1.** Amino acid decarboxylation assay

| **Strain** | **Time (h)** | | | | | | | | | | | | | | | | | | | | | | | | | | | | | |
| --- | --- | --- | --- | --- | --- | --- | --- | --- | --- | --- | --- | --- | --- | --- | --- | --- | --- | --- | --- | --- | --- | --- | --- | --- | --- | --- | --- | --- | --- | --- |
|  | **12 hours** | | | | | **24 hours** | | | | | **36 hours** | | | | | **48 hours** | | | | | **60 hours** | | | | | **72 hours** | | | | |
|  | **Arg.** | **His.** | **Lys.** | **Tyr.** | **Tryp.** | **Arg.** | **His.** | **Lys.** | **Tyr.** | **Tryp.** | **Arg.** | **His.** | **Lys.** | **Tyr.** | **Tryp.** | **Arg.** | **His.** | **Lys.** | **Tyr.** | **Tryp.** | **Arg.** | **His.** | **Lys.** | **Tyr.** | **Tryp.** | **Arg.** | **His.** | **Lys.** | **Tyr.** | **Tryp.** |
| PCFF-1 | N | N | N | N | N | N | N | N | N | N | P | N | N | N | N | P | N | N | N | N | P | N | N | N | N | P | N | N | N | N |
| Lp-UCC | N | N | N | N | N | N | N | N | N | N | P | N | N | N | N | P | N | N | N | N | P | N | N | N | N | P | N | N | N | N |
| Ls-UCC | N | N | N | N | N | N | N | N | N | N | P | N | N | N | N | P | N | N | N | N | P | N | N | N | N | P | N | N | N | N |
| R0011 | N | N | N | N | N | N | N | N | N | N | N | N | N | N | N | N | N | N | N | N | N | N | N | N | N | N | N | N | N | N |
| R0052 | N | N | N | N | N | NC | N | N | N | N | NC | N | N | N | N | NC | P | N | N | N | NC | P | N | N | N | NC | P | N | N | N |
| Lr-32 | N | N | N | N | N | N | N | N | N | N | N | N | N | N | N | N | N | N | N | N | N | N | N | N | N | N | N | N | N | N |
| Lpc-37 | N | N | N | N | N | N | N | N | N | N | N | N | N | N | N | N | N | N | N | N | N | N | N | N | N | N | N | N | N | N |
| Shirota | N | N | N | N | N | N | N | N | N | N | N | P | N | N | N | N | P | N | N | N | N | P | N | N | N | N | P | N | N | N |
| MXVK29 | N | N | N | N | N | P | N | N | N | N | P | N | N | N | N | P | N | N | N | N | P | N | N | N | N | P | P | N | P | N |
| DSM17918 | N | N | N | N | N | N | N | N | N | N | P | P | N | N | N | P | P | N | N | N | P | P | N | N | N | P | P | N | N | N |
| DSM17938 | N | N | N | N | N | N | N | N | N | N | P | P | N | N | N | P | P | N | N | N | P | P | N | N | N | P | P | N | N | N |

Arg: arginine; His: histidine; Lys: lysine; Tyr: tyrosine; Tryp: Tryptophan

P: decarboxylating strain of the amino acid used; N: non-decarboxylating strain of the amino acid used; NC: strain without growth in the medium.
